# Supplementary figures and images for: Model-based inference of neutralizing antibody avidities against influenza virus
Source: PLoS Pathog. 2022 Jan 31;18(1):e1010243. doi: 10.1371/journal.ppat.1010243 (PMC8830794; doi:10.1371/journal.ppat.1010243)

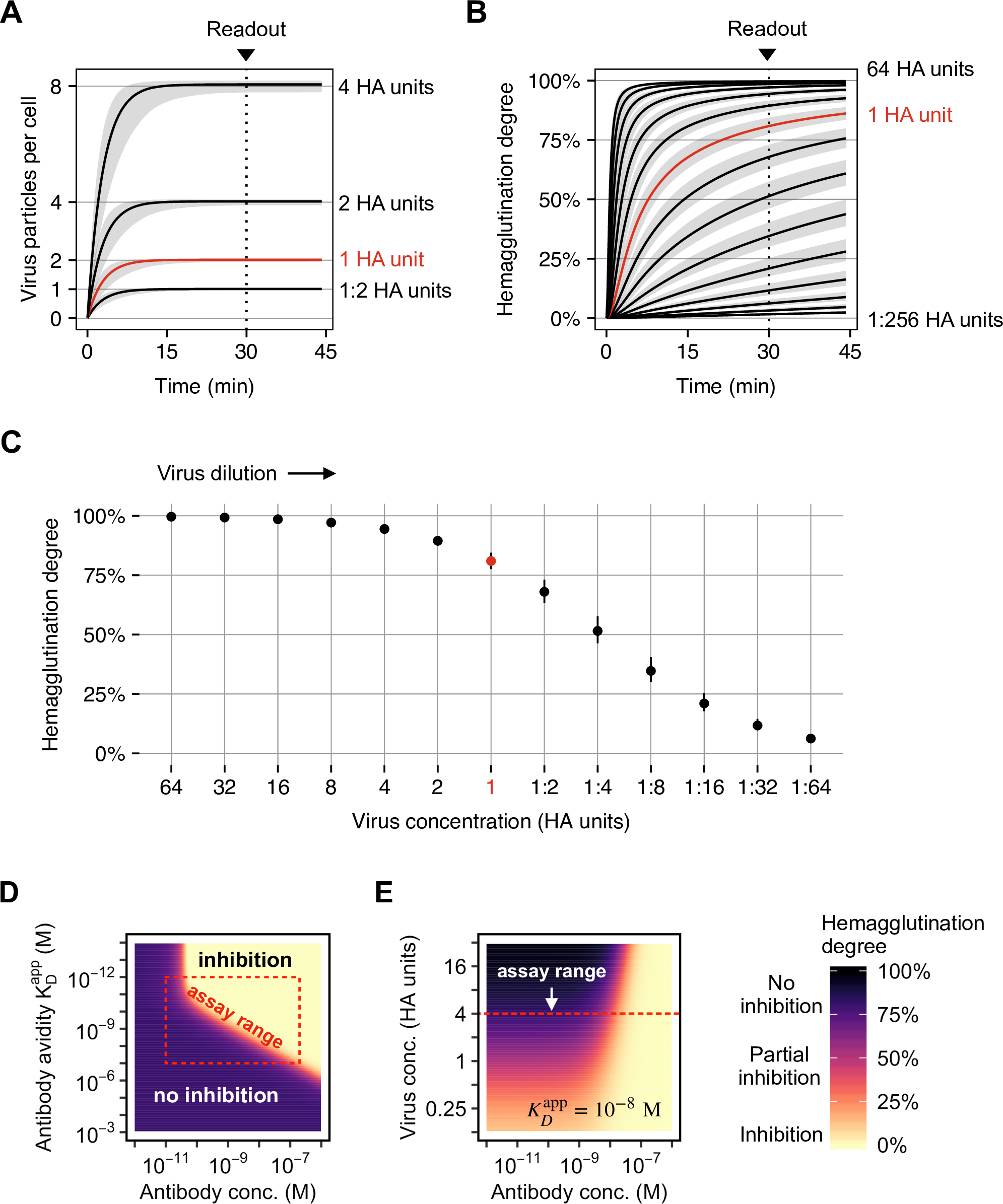

Supplement: S1 Fig — (A) Binding kinetics of virus particles to red blood cells. We assume that full hemagglutination requires at least two bound virus particles per cell. (B) Hemagglutination kinetics. (C) For HA units ≥ 1, the hemagglutination degree is > 75%, which is by definition interpreted as full hemagglutination. Gray areas and error bars indicate the uncertainty due to uncertainty in model parameters. (D) Performing the HI assay with 4 HA units balances sensitivity and robustness. There is a clear distinction between inhibition and no inhibition. (E) In addition, the assay detects with 4 HA units lower antibody concentrations than with ≥ 8 HA units. (TIF) [file ppat.1010243.s001.tif]

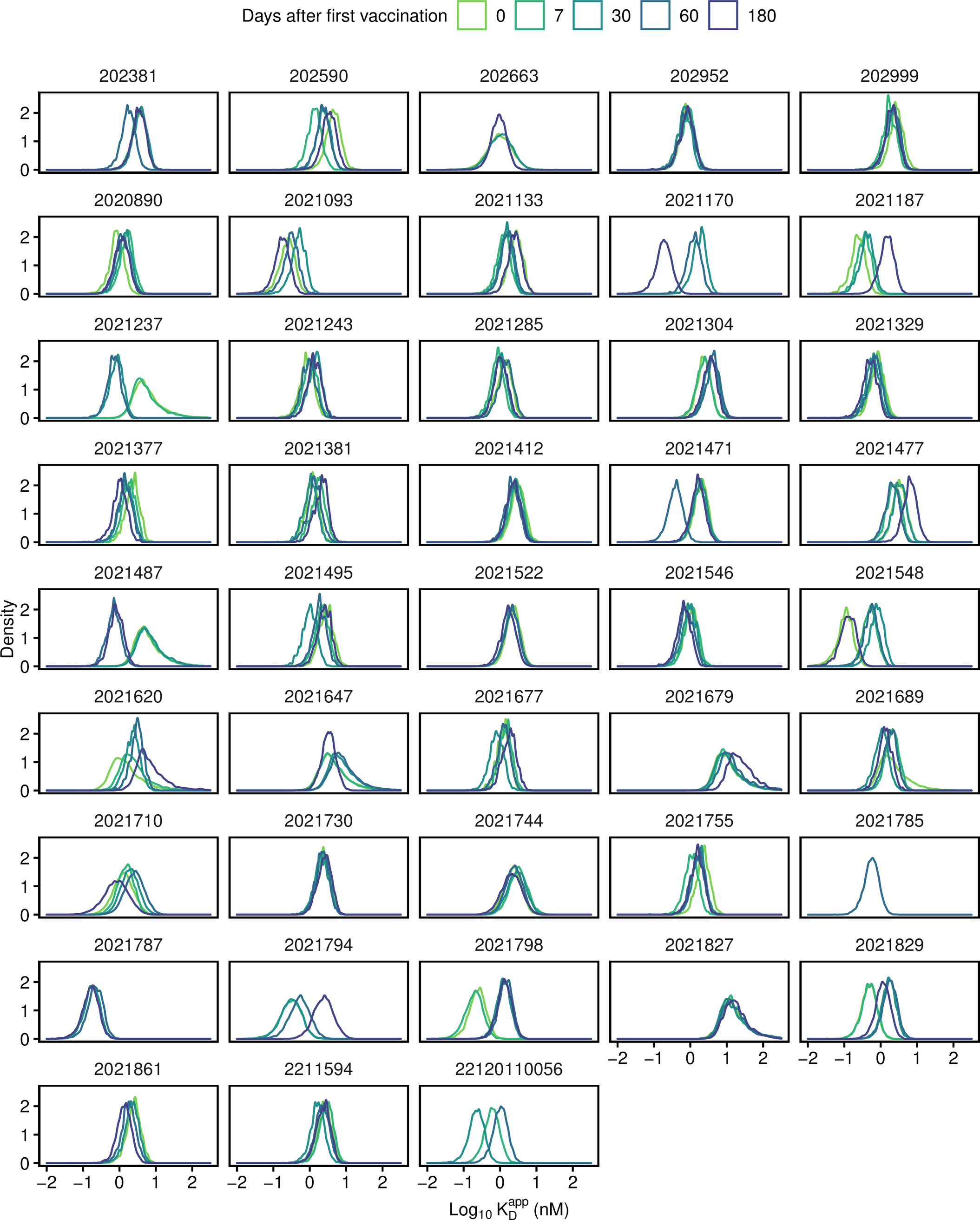

Supplement: S2 Fig — Some posteriors show larger variance due to larger measurement error in ELISA-detected IgG concentration. Here, for samples with HI titer < 8, the shown posterior distributions correspond to the inferred avidity when assuming HI titer = 4 (affected 23 serum samples from seven patients). (TIF) [file ppat.1010243.s002.tif]

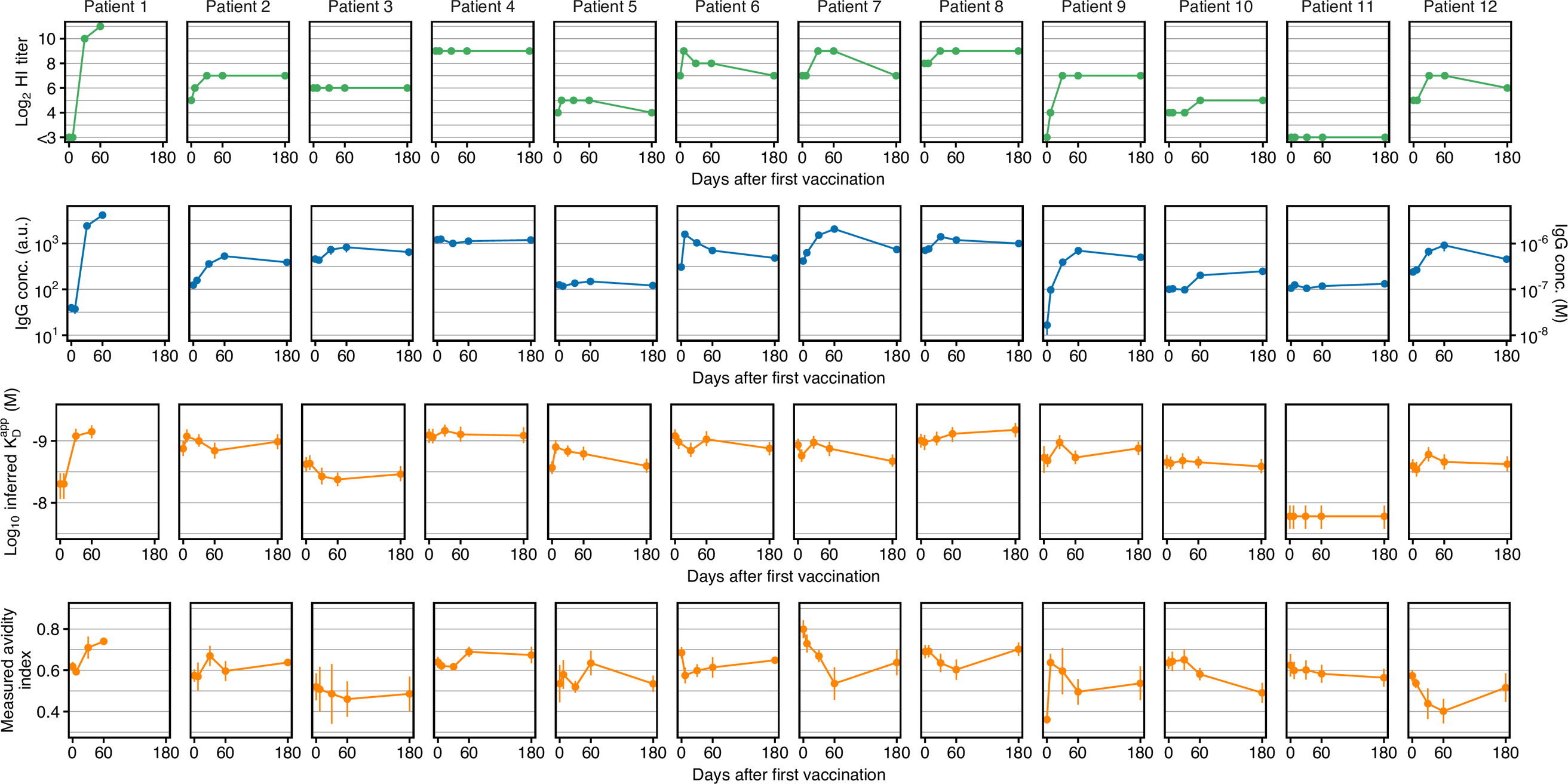

Supplement: S3 Fig — Avidity indices correspond to the fraction of H1N1pmd09-specific serum IgG remaining bound after 4M urea treatment. Data show mean and standard deviation for serum IgG and avidity indices and the median of the posterior distribution with the uncertainty range due to discretized HI titer measurements and ELISA measurement error for inferred apparent dissociation constants KDapp. Most patients showed either little or no increase in avidity. In some patients, the measured avidity decreased and then returned back to baseline on d180, potentially because the vaccine-induced short-lived antibodies were more sensitive to urea treatment, resulting in antibody denaturation. (TIF) [file ppat.1010243.s003.tif]

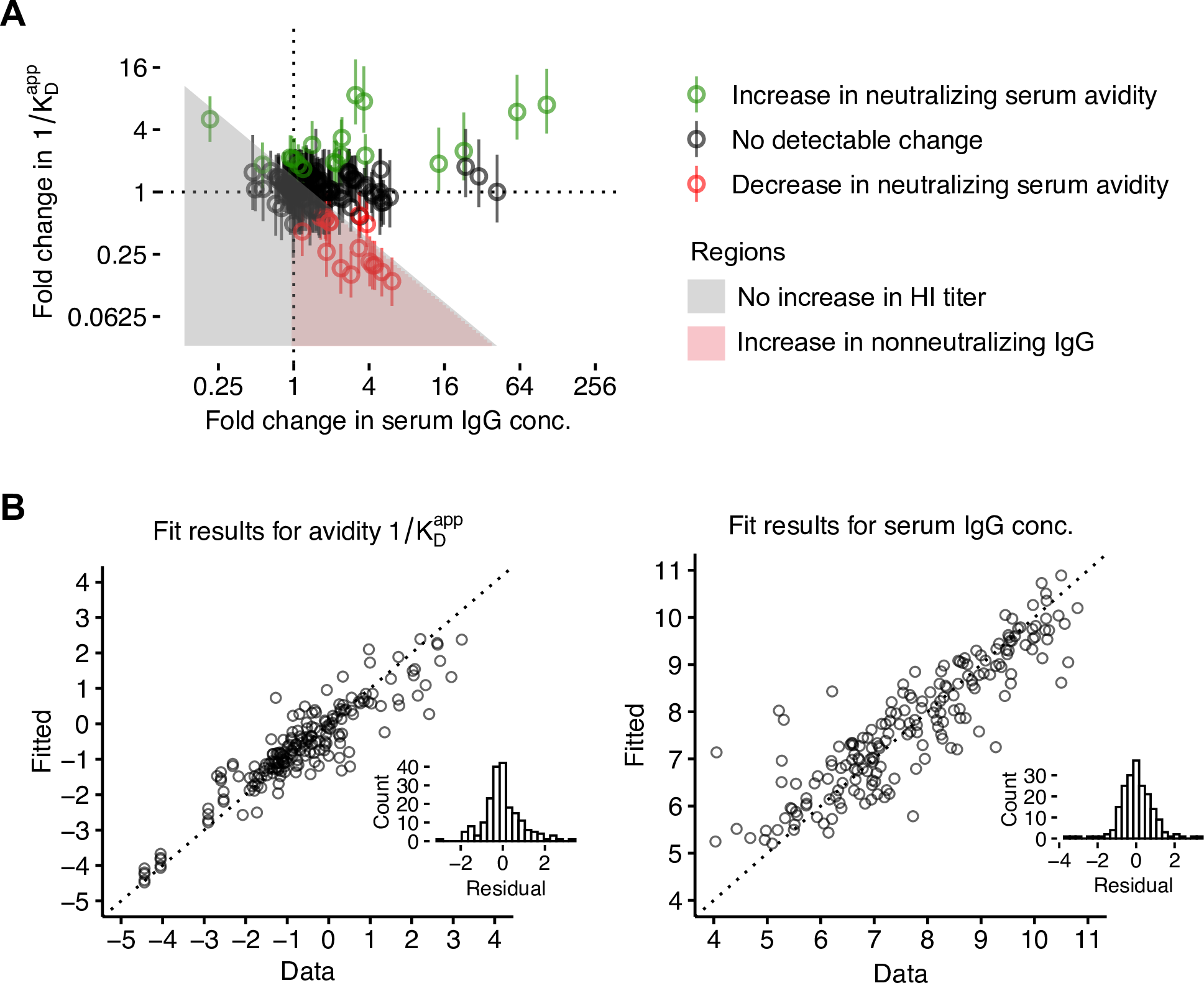

Supplement: S4 Fig — (A) Fold change in inferred avidity and serum IgG concentration after vaccination. Error bars indicate uncertainty in fold change due to uncertainty in inferred KDapp-values. Shading indicates regions with qualitatively different responses to vaccination. (B) Residual plots of the regression models used to investigate associations of criteria for compromised immune response with avidity and serum IgG concentration. (TIF) [file ppat.1010243.s004.tif]

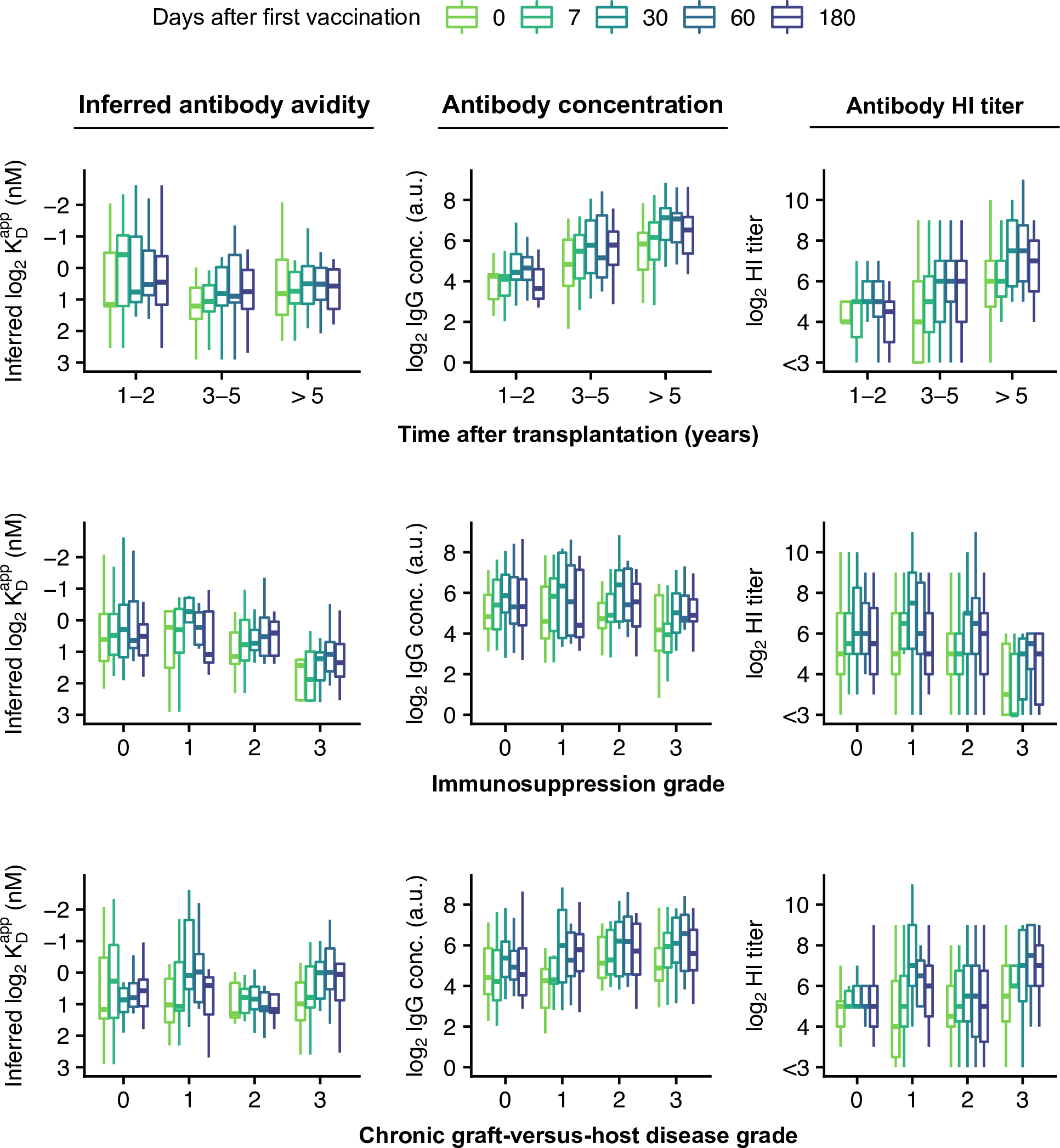

Supplement: S5 Fig — Note that data show one-dimensional associations, whereas regression analysis was performed with a high-dimensional model simultaneously accounting for time after transplantation, immunosuppression/cGVHD grade, and correcting for sex and age. (TIF) [file ppat.1010243.s005.tif]
