# Supplementary material for: Model-based inference of neutralizing antibody avidities against influenza virus
Source: PLoS Pathog. 2022 Jan 31;18(1):e1010243. doi: 10.1371/journal.ppat.1010243 (PMC8830794; doi:10.1371/journal.ppat.1010243)
Supplement: Attachment — Submitted filename: hi-manuscript_response_letter.pdf [file ppat.1010243.s006.pdf]

Response Letter PPATHOGENS-D-21-01601
Case Number: 07378525
Manuscript Title: Model-based inference of neutralizing antibody avidities against influenza virus
Authors: Janina Linnik, Mohammedyaseen Syedbasha, Yvonne Hollenstein, Jörg Halter, Adrian Egli, Jörg Stelling
Correspondence to: joerg.stelling@bsse.ethz.ch

We thank all reviewers for their thoughtful and constructive comments. Please find our point-by-point answers below. The line numbers refer to the revised manuscript version with the highlighted revisions.

Major Comments

Review #2

1. To establish their model, the authors generated an HI 'training' dataset using a monoclonal HA antibody and the 2009 pandemic H1N1 strain, and various parameters from the literature, such as the sialic acid-HA binding constant of the A/X-31 H3N2 strain. The authors claim that they used these parameters to "make the model more specific for H1N1 pdm09" (line 94/95). However, the H3N2 virus (note that X-31 is a recombinant A/Aichi/1968 virus) is a rather different virus than the pandemic H1N1 virus. Moreover, HA proteins from different influenza virus strains can have widely different Kd's for different sialic acids and bind sialic acid in different ways (e.g. }). These very basic points makes one wonder if the authors should not have spent some effort on characterizing the biochemical and biophysical properties of their HA and antibody combination, before making sweeping claims about the general applicability and robustness of their model.

Thank you for sharing this reference. We agree that the sentences in **lines 94/95** were not properly reflecting our rationale and we updated several sections accordingly (**lines 94-99**, **lines 112-118** and **lines 144-149**):

First, we did not train the model on a training data set from a monoclonal antibody. Instead, we extracted from the literature all parameters (and their biologically reasonable uncertainty ranges) that are related to chicken red blood cells (RBCs) and influenza virus except for the agglutination rate constant, which is unknown. This constant describes the agglutination of RBCs to macroscopic aggregates (due to cross-linking of RBCs via virus particles with free HA receptors). We inferred this parameter from independent hemagglutination inhibition experiments with a serum sample from a healthy volunteer (**cf. Methods, revised lines 491-493**).

To identify a suitable HA-sialic acid binding constant, we investigated a broad, biologically reasonable range of potential sialic-acid-HA binding constants as follows: since the association and dissociation rate constants for the A/X-31 H3N2 strain were well-characterized, we first

used these values as a reference for a biologically reasonable order of magnitude for the binding constant of HA to sialic acid (corresponds to a dissociation constant of approximately 30 nM to 300 nM). We then investigated in a sensitivity analysis the impact of varying sialic-acid-HA binding constants on our primary model outcome (i.e., the hemagglutination degree) as now described in **lines 112-118.** We sampled the corresponding association and dissociation rate constants from log-normal distributions (**Table 1**), which correspond to a log-normally distributed dissociation constant centered at 100 nM and covering a range of approximately 30 nM to 300 nM. Our sensitivity analysis shows that the uncertainty in this binding constant has only little influence on the hemagglutination degree (**Figure 2a**). The intuitive explanation of this observation is that the number of sialic acid receptors is approximately two orders of magnitudes larger than the number of HA receptors under assay conditions (**Table 1**). This leads almost to saturation of free HA receptors after 30 min incubation and little sensitivity to varying binding constants (if the binding constant is in the assumed range).

Our claim about the robustness of the model refers to the sensitivity analysis results presented in **Figure 2a**. These results show that the model is robust to uncertainty in model parameters (or parameter assumptions) specific to RBCs and virus particles as well as to the variability in experimental conditions. We have updated **lines 94-99**, **lines 112-118** and **lines 145-149** to make this more clear.

2. When the authors use the model to infer the antibody concentration and avidity from patient samples, they fix the model parameters (line 140-141). It is unclear why they chose this approach, given that their model was trained using a monoclonal antibody and parameters that may not be appropriate for all serum samples. No experimental data is shown to justify this approach.

Thank you for your comment, we have updated several lines accordingly (**lines 155-157**). First, we did not fix all model parameters, but only the parameters that are specific to RBCs and virus particles since all serum samples were performed under the same assay conditions. The parameters that are specific to serum samples, i.e., the apparent serum antibody avidity and antibody concentration, were inferred for each serum sample individually. Second, the model was not trained on a monoclonal antibody (as also described above). Instead, RBC- and virus-specific model parameters (and their biologically reasonable uncertainty ranges) were extracted from the literature except for the agglutination rate constant. The agglutination rate constant was estimated from hemagglutination inhibition experiments with serum samples of a healthy volunteer (**cf. Methods, revised lines 491-493**). Since this constant describes the interaction of RBCs and free HA-receptors on virus particles, we assume that it does not depend on the serum sample (our model neglects any unspecific binding of antibodies, cf. **lines 63-65)**.

We infer apparent antibody avidities from HI titers and ELISA-detected antibody concentrations under the assumption that the observed differences in hemagglutination degree between serum samples result solely from differences in apparent serum antibody avidity and antibody concentration. There are two justifications: first, all samples were measured under the same

assay conditions, implying all parameters related to assay conditions, RBCs and virus particles should be identical. The second and more important justification is that we explored in a sensitivity analysis how the uncertainty in model parameters (or parameter assumptions) specific to RBCs and virus particles as well as the variability in experimental conditions (**lines 102-118** and **lines 145-149**) affect the hemagglutination degree (as also described above). The results in **Figure 2a** show that the variability in hemagglutination degree is mostly explained by the variabilities in serum antibody concentration and apparent antibody avidity -- when assuming a biologically reasonable range for antibody concentration, avidity and all other model parameters as summarized in **Table 1**. In summary, variability in model parameters specific to RBCs and virus particles contributes only little to the observed variability in hemagglutination degree. Therefore, we conclude that the variability in hemagglutination degree between serum samples can be mostly explained by the variability in apparent serum antibody avidity and concentration. We modified the corresponding section to clarify that the sensitivity analysis is the justification for fixing the remaining model parameters when we estimate the apparent antibody avidity from antibody concentration and HI titer (**lines 154-157**).

Reviewer #3

Line 218-225: Authors stated that the patients with HSCT > 2 years may have developed durable H1N1pmd09-neutralizing antibody; whereas the early transplant patients (HSCT < 2 years) with lower IgG concentration and HI titer was resulted in previous vaccinations did not induce (detectable) affinity maturation. However, early transplant patients might also receive vaccines before receiving a transplant.

Yes, this is correct. All HSCT patients were probably in contact with influenza before transplantation (through vaccination or infection). However, to enable engraftment of hematopoietic stem cells, patients have to undergo chemotherapy and other treatments that severely deplete their immune system before transplantation. Immunological memory acquired before transplantation is therefore extremely limited.

Unfortunately, the patients' immunization history before and after transplantation was unknown and we could not correct for this factor in the statistical analysis. We added a sentence to highlight this limitation (**lines 243-245**).

In HSCT < 2 years patients, the vaccine-induced immunity may also be compromised by the onset of immunosuppressive treatment. These descriptions could be moved to the discussion section

Yes, this is correct because early transplant patients are more likely to receive immunosuppressive treatment. Therefore, we performed a multivariable regression analysis, where we corrected for the immunosuppression grade (and also for cGVHD grade, sex and age). Thus, the inferred effect of time after HSCT (<=2 years vs >2 years) is independent of immunosuppression grade. We revised the corresponding lines to make this more clear (**lines 236-238**).

In our multivariable analysis, we could not detect a significant effect of immunosuppression grade on the baseline levels in antibody concentration but on the baseline levels in antibody avidity (**lines 245-249**).

Minor Comments

Reviewer #1: The manuscript is generally straightforward – however there are other limitations to this as contemporary H3N2 viruses are known to have agglutination problems and thus this approach is arguably effective only when assessing the response towards H1N1s. Perhaps this should be briefly discussed or pointed on in the discussion.

Yes, our model is only applicable if the virus strain shows hemagglutination or, more generally, where the hemagglutination inhibition assay yields informative results. We have now highlighted this limitation in the discussion as suggested (**lines 271-274** and **lines 279-281**).

Reviewer #2: Minor comments:

3. The authors use the output of the model to assess the quality of the model in Fig. 2. However, the authors do not use experimental data to verify the output of the model at this point. Indeed, Fig. 2b and c only show predicted data, but no comparison to experimental data and statistical testing is used to demonstrate how reliable the model is. It is thus unclear why the authors call their model "robust" (line 111 and 134). The comparison with the experimental data follows in Fig. 3, so these claims seem premature at this point in the text.

Our claim about the robustness of the model refers to the sensitivity analysis results presented in **Figure 2a**. These results show that the model is robust to the uncertainty in model parameters (or parameter assumptions) specific to RBCs and virus particles as well as to variability in experimental conditions (**lines 145-149**). However, we are aware that the term "robustness" is frequently used to characterize the ability of a model to predict new data. Here, we refer to robustness to explain the relationship between model inputs and model output (**lines 102-107**). We have modified the language in this section and added additional explanations for clarification (**lines 94-99**, **lines 112-118** and **lines 145-149**).

4. line 64: The authors report that they use a protease treatment of the serum sample to minimize unspecific antibody binding. This protease treatment is confusing, because it is not explained in the material and methods section. Instead, the authors describe treating the serum with a sialidase, which is a glycoside hydrolase and not a protease at all. What is the method that they used?

Thank you for pointing out this inconsistency. We have changed the word "protease" to "receptor destroying enzyme" (**lines 64/65**). Serum samples were pre-treated with Cholera filtrate (Sigma-Aldrich #C8772), which contains neuraminidase/sialidase.

5. Figure 4c is not referenced in the main text.

It was erroneously referenced as Figure 3c, we have corrected it (**line 216**).

Reviewer #3: -As the authors described in Methods (line 315-316) and line 142-143, all patients were immunized with two doses of trivalent seasonal influenza vaccine. The manuscript only presents specific immune response against H1N1pmd09. Please provide some discussion about the inferences of antibody avidity against other virus strains.

In general, the model is only applicable to virus strains that show hemagglutination activity. We have added several lines to our discussion to point out which aspects would need to be considered for other virus strains (lines **271-274** and lines **279-281**).

-In line 241-244, the authors stated that results from the model explained why the HI assay is the gold standard in serological studies and the limitations of the current model. In line 249-257, the authors also mentioned some limitations of the model and provide some resolutions for these limitations. Accordingly, it still needs techniques (SPR or calorimetry measurements) to overcome these limitations. The authors should provide some discussion to emphasize the advantages and impacts of the biophysical model in future vaccine studies.

Current techniques for measuring avidity are time consuming and expensive, either because they require special equipment, tedious protocol optimization, or both. Our model-based approach allows to infer avidities from easy-to-acquire measurements with a precision of approximately +/- 30%. Thereby, it can detect candidates for affinity maturation or non-neutralizing antibody production. We see the main advantage of this approach in enabling rapid screening of serum samples on a large scale to detect outliers or candidates for affinity maturation. The samples of the candidates of interest can then be further investigated by SPR or calorimetry for confirmation. We have added corresponding statements to the Discussion (**lines 317-319**).
